# CGMacros: a pilot scientific dataset for personalized nutrition and diet monitoring

**DOI:** 10.1038/s41597-025-05851-7

**Published:** 2025-09-25

**Authors:** Anurag Das, David Kerr, Namino Glantz, Wendy Bevier, Rony Santiago, Ricardo Gutierrez-Osuna, Bobak J. Mortazavi

**Affiliations:** 1 Texas A&M University, College Station, 77843 USA; 2 Sutter Health, Santa Barbara, 93101 USA; 3 Health Linkages, Santa Barbara, 93110 USA; 4 Sansum Diabetes Research Institute, Santa Barbara, 93105 USA

**Keywords:** Metabolic disorders, Biomedical engineering

## Abstract

Tracking food intake is key to using nutrition to prevent or manage common diseases including type 2 diabetes (T2D) and obesity. Several datasets are publicly available to promote research in diet monitoring, but generally contain data from a limited set of sensors (e.g., accelerometry, food images), which limits their application to specific use cases such as activity recognition or image recognition. Also lacking are publicly available datasets with food macronutrients and their associated continuous glucose measurements; datasets containing such rich information are proprietary. To address this gap, we present CGMacros, a dataset containing multimodal information from an activity tracker, two continuous glucose monitors (CGM), food macronutrients, and food photographs, as well as anonymized participant demographics, anthropometric measurements and health parameters from blood analyses and gut microbiome profiles. CGMacros contains data for 45 participants (15 healthy, 16 pre-diabetes, 14 T2D) who consumed meals with varying and known macronutrient compositions in a free-living setting for ten consecutive days. To our knowledge, this is the first database of its kind to be made publicly available. CGMacros, and larger publicly available datasets that we hope may follow, are essential to democratize academic research in personalized nutrition and algorithmic approaches to automated diet monitoring.

## Background and related datasets

Poor dietary habits are a major contributor to the development of chronic diseases such as type 2 diabetes, obesity, heart disease, and some cancers. A recent survey examining food consumption across 195 countries estimated that improving diet can prevent one of every five deaths worldwide^1^. Therefore, monitoring food intake is an important step towards maintaining a healthy diet and preventing chronic diseases later in life. Current methods for diet monitoring are based on self-report measures (e.g., food journals and mobile apps). Such methods allow users to track their eating habits, adhere to a diet plan, and reflect on situations when they stray away from their plans. However, these methods often require prolonged manual entry, which is cumbersome and can lead to errors, or barcodes, which may promote consumption of processed foods^2^.

The advent (and increased prevalence) of wearable sensors has led to the development of automated methods to detect and recognize moments of food intake. These sensors include smartwatches and smart utensils with embedded accelerometers that track hand-to-mouth gestures as a proxy for detecting eating instances^3,4^. Datasets based on these technologies have been publicly released to advance the field of nutrition monitoring. As an example, the Food Intake Cycle (FIC) dataset^5^ contains accelerometer data with annotated start and end meal instances from 21 meal sessions and 12 unique subjects in a cafeteria setting. The original dataset has been extended to include information from participants eating in free living conditions^6^. Additional sensing modalities, such as audio recordings can also be used to detect sounds associated with food intake, such as chewing and swallowing. This type of data is also publicly available. As an example, the audio-based calorie estimation (ACE) dataset^7^ contains accelerometry and audio recordings from seven participants wearing sensors on their head and wrist, and annotations of the amount and type of food and drinks from video feed recorded using a Google Glass. The Clemson Cafeteria dataset^8^ contains data from multiple sensors (wrist motion, a scale embedded on a food tray, and synchronized video cameras recordings from overhead cameras and chest-worn cameras) on a larger set of 264 participants. Similarly, the OREBA dataset^9^ contains inertial measurement unit (IMU) readings (accelerometer and gyroscope) for both hands and synchronized frontal videos of 100 participants consuming a discrete dish and 102 participants sharing a dish, with a total of 9,069 intake gestures. Given the pervasiveness of accelerometers in wearable devices, these types of datasets can support the development of technological approaches to nutrition monitoring at scale, but inertial measurements are restricted to detecting eating moments.

The widespread use of smartphones has also made it possible to log food choices by simply taking a photograph, which opens a broad range of possibilities in free-living conditions -when compared to worn or ambient cameras in controlled environments, which may raise privacy concerns. Modern machine/deep learning techniques can be used to analyze food photographs. Several datasets have been publicly released for this purpose, such as UECF Food 100^10^, FoodX-251, and Food2K^11^, which contain thousands of images with diverse food items and the corresponding nutrition labels. However, extracting accurate estimates of food macronutrients and caloric content is challenging given the variety of ways in which foods can be prepared (e.g., oil amounts, fat content). To address this gap, the Recipe1M^12^ dataset contains one million structured cooking recipes and their corresponding images and has been used extensively for food recognition. The closely-related Nutrition5k^13^ dataset also contains annotated nutritional information of over 5000 diverse, real-world food dishes along with the food photographs.

In prior work^14,15^, we have shown that CGMs may be used to estimate food macronutrients, avoiding the need for manual annotation or food photography, though the latter can provide complementary information to glucose measurements^16^. Since postprandial glucose responses (PPGR) depend not only on carbohydrate content of a meal but also the amounts of protein and fat, analyzing the shape of a PPGR with machine learning (ML) models can provide estimates of a meal’s macronutrient content. At present, however, CGM datasets with macronutrient information are very limited, restricting their use to prediction of hypo/hyperglycemia events in patients with type 1 diabetes (T1D). Several datasets are publicly available for this purpose, including the OhioT1DM^17^, D1NAMO^18^, and T1DiabetesGranada^19^ datasets. The remaining datasets that we are aware of (ARISES, ABC4D) are not publicly available^20^. Only recently, a dataset of 102 Chinese participants (12 with T1D, 100 with T2D) patients was publicly released^21^. The dataset contains 24-hour glucose data, food names and their quantity (e.g., boiled egg 40g, boiled vegetable 116g) but not the macronutrient composition of those foods.

To our knowledge, our proposed CGMacros dataset^22^ is the first to include CGM recordings with meal macronutrients, food photographs, physical activity (accelerometry data), and health and demographic parameters. Table 1 summarizes this information for CGMacros and other publicly available datasets.

**Table 1:** Summary of CGMacros^22^ and other existing publicly available datasets for diet monitoring, according to population health, availability of CGM recordings, food macronutrients, food photographs, physical activity, fasting insulin, mocriobiota, and experimental setting.

Dataset	Population	CGM	Macros	Images	Activity	Insulin	Microbiome	Setting
ACE^7^	NA	No	Yes	No	No	No	No	Controlled
OREBA^9^	NA	No	No	No	No	No	No	Controlled
UECF Food 100^10^	NA	No	No	Yes	No	No	No	Ambulatory
Nutrition5k^13^	NA	No	Yes	Yes	No	No	No	Ambulatory
OhioT1DM^17^	T1DM	Yes	No	No	No	Yes	No	Ambulatory
D1NAMO^18^	T1DM	Yes	No	No	No	Yes	No	Ambulatory
Zhao *et al*.^21^	T1/T2DM	Yes	Yes	No	No	Yes	No	Ambulatory
**CGMacros**^22^	T2DM*	Yes	Yes	Yes	Yes	Yes	Yes	Ambulatory

* Includes healthy adults and adults with pre-diabetes.

## Methods

Participants for the study were recruited at Sansum Diabetes Research Institute (SDRI), in Santa Barbara, CA. On day 1 of the study, potential participants cleared an initial screening and signed a consent form (Advarra IRB Pro00049227; ClinicalTrials.gov NCT04991142). As part of the screening process, we measured the participant’s body mass index (BMI), glycated hemoglobin (HbA1c), fasting glucose, fasting insulin, triglycerides, and cholesterol levels. At this time, we also recorded their demographic information (age, gender, and race). Exclusion criteria for subjects with type 2 diabetes (T2D) was being treated with oral medicines (other than Metformin) or any injectable GLP-1 receptor agonist or insulin. After the initial screening, an Abbott FreeStyle Libre Pro CGM (15-min sampling period) and a Dexcom G6 Pro CGM (5-min) were placed on the participant’s upper arm and abdomen, respectively. Both CGMs were blinded to prevent glucose readings from influencing participants. Participants were also provided with a Fitbit smartwatch (Fitbit Sense) to log exercise, and were trained to use the MyFitnessPal mobile app to log their meals and take pictures of their foods using the WhatsApp mobile app.

Forty-five participants completed the study, ages 18-69, and body mass index (BMI) 21-46 kgm^2^. Table 2 summarizes the demographic information of study participants. All participants were recruited between 2021 and 2024. Out of 45 participants, 15 had no pre-existing diabetes (*HbA*1*c* < 5.7%), 16 had pre-diabetes (5.7≤*HbA*1*c*≤6.4%), and 14 had type 2 diabetes (T2D) (*HbA*1*c* > 6.4%).

**Table 2:** Summary of demographic information of all patients in the CGMacros dataset^22^.

Characteristics	Measurement
Age (years)	48.11 ± 12.70
Self-reported gender (male/female)	16/29
BMI (kg/m^2^)	31.15 ± 6.65
Race (White/Hispanic or Latino/African American)	7/34/4
HbA1c (%)	6.16 ± 0.91
Healthy/Pre-diabetes/Type 2 diabetes	15/16/14
Fasting glucose (mg/dL)	120.69 ± 30.23
Fasting insulin	14.43 ± 8.16

Data are presented as mean ± standard deviation (SD) or number of subjects in the group. BMI: body mass index; HbA1c: glycated hemoglobin, a measure that correlates with the average glucose levels over the previous 2-3 months.

Each subject recorded their meals for 10 days, including breakfast, lunch and dinner. Breakfasts consisted of protein shakes with varying amounts of carbohydrates, protein, fat, and fiber. Lunches were ordered from a local, fast-casual restaurant chain (Chipotle Mexican Grill). The breakfast and lunch meals were designed to cover a range of macronutrient contents -see Tables 3 and 4. For dinners, participants ate foods of their own choice. To minimize interferences in glucose responses from prior meals, participants were instructed to eat lunch at least 3 hours after breakfast, with only water or coffee (without sugar) in between, and dinner at least 3 hours after lunch. They also took photographs of the meals before and after eating, from which we extracted the meal timestamps and the proportion of the meal they consumed. Stool samples were collected at the start of the study and analyzed using a Viome microbiome kit (Viome Life Sciences, Inc.).

**Table 3:** Macronutrient composition of breakfast shakes.

Study day	Meal	Carb (g)	Protein (g)	Fat (g)	Fiber (g)
1	HLLL	66	22	11	00
2	HHLL	66	66	11	00
3	HLHL	66	22	42	00
4	HHHH	73	66	42	07
5	LLLL	24	22	11	00
6	HLLL	66	22	11	00
7	HHLL	66	66	11	00
8	HLHL	66	22	42	00
9	LLLL	24	22	11	00
10	HLHH	66	22	42	07

Meals are coded as having high (H) or low (L) macronutrient composition based upon US average daily intake of carbs, proteins, fat, and fiber, respectively^35^. For example, HLLL denotes a meal high in carbohydrates and low in protein, fat and fiber, whereas HHLL denotes a meal high in carbohydrates and protein, but low in fat and fiber.

**Table 4:** Macronutrient composition of lunch meals.

Study day	Meal	Carbs (g)	Protein (g)	Fat (g)	Fiber (g)
1	HHHH	81	88	54.5	18
2	HLHL	92	17	42	10
3	LHLL	16	66	14	4
4	HLLL	94	12	13	5
5	LLLL	19	32	15	5
6	HHHL	93	84	44	4
7	HLLH	76	22	18.5	11
8	LHLH	40	76	17	13
9	LLLH	43	20	20	13
10	HHLL	94	44	20	4

Meals are coded as having high (H) or low (L) macronutrient composition based upon US average daily intake of carbs, proteins, fat, and fiber^35^.

**Fig. 1:**
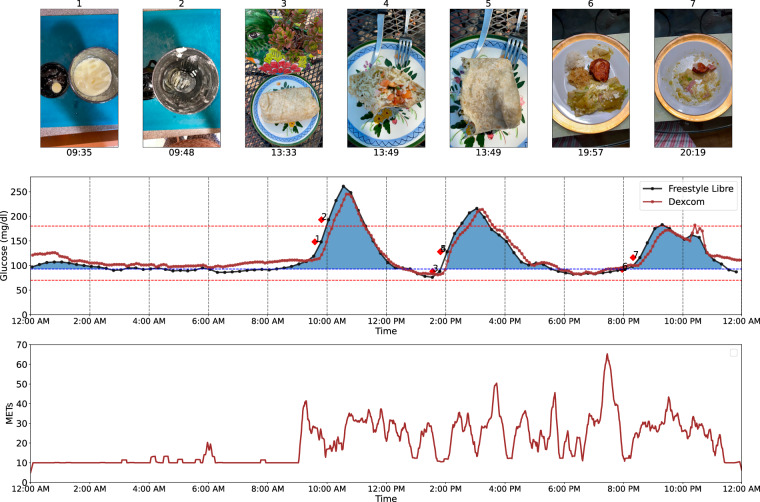
Food photographs, CGM recordings and physical activity of a study participant over a 24-hour period. Note that FitBit reports the value of activity as METs multiplied by 10.

To illustrate the type of postprandial glucose responses (PPGRs) to meals, Fig. 1 shows the glucose profile of the two CGM devices along with meal and exercise information in metabolic equivalent of tasks (METs) over a 24-hour period for one participant. Red markings atop the CGM curve denote times at which meals started and ended. Also shown are food photographs that the participant sent via WhatsApp. The timestamp of those photographs was extracted from WhatsApp as well. To compute METs we used measurements from the Fitbit device provided on a minute-by-minute basis and applied a mean filter with a window size of 20 minutes.

## Data Records

All data records in the dataset are accessible on figshare^22^. The dataset consists of 45 main CSV files (**CGMacros-#.csv**), where # denotes participant number, and three supplementary CSV files. The main files contain CGM and fitness tracker readings, one row per measurement (plus a heading), and one column per variable. Since the Abbott and Dexcom CGM devices have different sampling rates, we used linear interpolation to obtain a uniform sampling rate of one minute. For the Abbott FreeStyle Libre Pro, we took the two consecutive CGM readings separated by 15 minutes and linearly interpolated between them to obtain CGM readings every minute. Similarly, for the Dexcom G6 Pro, we linearly interpolated between consecutive readings separated by 5 minutes. We also report heart rate and MET from Fitbit at one-minute intervals. At the appropriate time stamp (i.e., row) from WhatsApp photographs, we also report the total caloric content and carbohydrate, protein, fat, and fiber amounts of each meal, the type of meal (breakfast, lunch, dinner) and a path to the file containing the corresponding photograph. Data from each participant spans approximately ten days.

The supplementary spreadsheet (**bio.csv**) contains demographics (age, gender, ethnicity), anthropometric measurements (height, weight, BMI), blood analytics (HbA1c, fasting glucose, insulin, triglyceride, cholesterol, high-density lipoprotein (HDL), non-HDL, low-density lipoprotein (LDL), very low-density lipoprotein (VLDL) levels, three finger stick glucose measurements and microbiome profile, all taken on the first day along for each study participant, with the corresponding date and time stamp. For each participant, Viome provides two reports, the first one listing all the bacteria that are present in the stool sample, and the second one providing digestive health scores and recommendations that Viome generates (recommendations are not included in this dataset). We combined the Viome reports of bacteria of the 45 participants and generated an indicator variable as a separate column for each of 1,979 bacteria, denoting whether it was present (1) or absent (0) in the corresponding Viome report. From the report of 22 gut health scores generated by Viome, we developed an ordinal variable for each of the tests coded as Good, Average, or Not Optimal. These scores are Viome’s estimate of gut health based upon the bacteria identified and include their estimates of overall gut health^23^. Examples of such scores include an overall Gut Health test, Metabolic Fitness, Inflammatory Activity, Digestive Efficiency, Gut Active Microbial Diversity, and summaries of present or non-present bacteria from the first report.

## Technical Validation

To establish the validity of the CGMacros dataset^22^, we report initial analyses of average glucose readings for each of the two CGM devices, stratified by metabolic status (healthy, pre-diabetes, T2D). We also provide an analysis of the time in range (TIR) per group and CGM device at two hyperglycemic thresholds (180 mg/dL, 250 mg/dL). Finally, we predict the 2-hr incremental area-under-the curve (iAUC) and absolute area under the curve (AUC) of the breakfast shakes from features derived from CGM, blood parameters, anthropometrics, and macronutrients and rank them in order of importance.

**Fig. 2:**
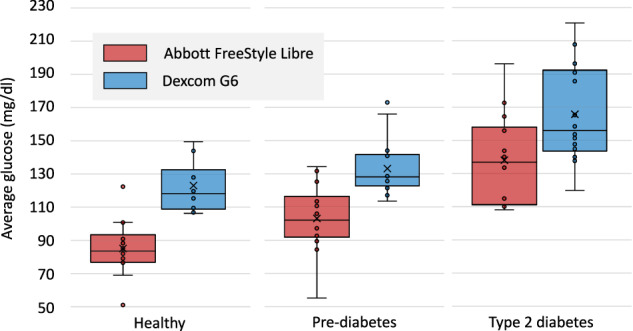
Glucose response for healthy adults, pre-diabetes and T2D for the Abbott FreeStyle Libre (red) and the Dexcom G6 (in blue) CGMs. Triangles indicate average glucose; solid lines denote median glucose.

Figure 2 summarizes the average glucose level across the ten study days, grouped by metabolic status. As expected, we observed a clear increase in average glucose levels when comparing healthy adults, and those with pre-diabetes and T2D -for both CGM devices. The average glucose from the Abbott Freestyle device was 84.89 mg/dL for healthy adults, 105.2 mg/dL (*p* < 0.01) for pre-diabetes, and 138.04 mg/dL (*p* < 0.01) for T2D. The average glucose from the Dexcom G6 device was 122.36 mg/dL for healthy adults, 135.1 mg/dL (*p* < 0.05) for pre-diabetes, and 165.7 mg/dL (*p* < 0.01) for T2D. We also found significant differences between the two CGM devices for each group: healthy (*p* < 0.001), pre-diabetes (*p* < 0.01), and T2D (*p* < 0.05), with Dexcom G6 glucose readings being higher by up to 58.7 mg/dl (for healthy adults). Inconsistencies between earlier generations of the two devices have been reported in the literature^24^, and may be related to the anatomical locations of the CGM (upper arm for Abbott vs. abdomen for Dexcom) and the corresponding differences in subcutaneous fat^24^.

**Fig. 3:**
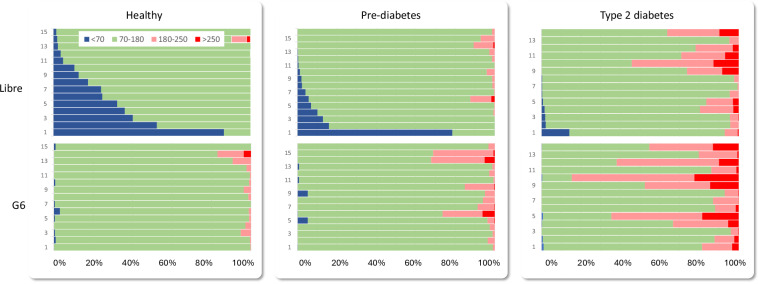
Time in range for individual participants in the three metabolic status groups (healthy, pre-diabetes, type-2 diabetes) for the Abbott FreeStyle Libre (top) and Dexcom G6 (bottom).

Figure 3 shows the proportion of time (i.e., CGM readings) in different glucose regions for participants, organized by CGM device and metabolic state. To analyze these results, we conducted a repeated-measures two-way ANOVA with CGM device and metabolic state as independent factors. For **time below range** (<70 mg/dL), we found main effects for device (*p* < 0.001) and state (*p* < 0.01), as well as interactions (*p* < 0.01). Post-hoc tests using Tukey’s Honest Significant Difference (HSD) found that time in hypoglycemia was significantly different between the two devices (*p* < 0.01) as well as between healthy and T2D groups (*p* < 0.05). For **time-in-range** (70-180 mg/dL; TIR), two-way ANOVA revealed a main effect for state (*p* < 0.01) and interactions (*p* < 0.01) but no main effect for device (*p* = 0.31). A post-hoc test using Tukey’s HSD found that the average TIR was significantly different between pre-diabetes and T2D groups (*p* < 0.05). For **time above range** (>180 mg/dl; %HG), we found a main effect for device (*p* < 0.001) and state (*p* < < 0.001) but no interactions (*p* = 0.18). Tukey’s HSD for multiple comparisons found that the average time in hyperglycemia was significantly different between the two sensors (*p* < 0.05) as well as between healthy and T2D groups (*p* < 0.01) and healthy and prediabetes groups (*p* < 0.01). Thus, these results indicate that the average time in hypoglycemia and hyperglycemia is influenced by metabolic status (as predicted) and by the CGM device (as previously reported^24^). However, for time-in-range the effect is only significant for metabolic state.

## Prediction of breakfast postprandial glucose responses

**Fig. 4:**
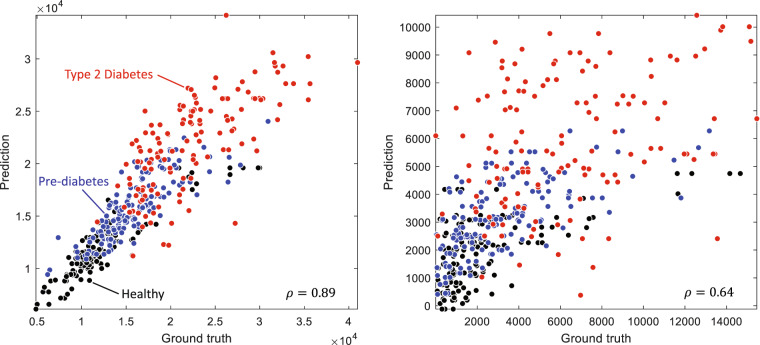
Correlation between (**a**) predicted and ground truth AUC, and (**b**) predicted and ground truth AUC.

As a final validation step, we built a machine-learning model to reproduce results in the personalized nutrition study by Zeevi *et al*.^25^. The model used anthropometric features, blood parameters, and macronutrient amounts to predict breakfast PPGRs. Anthropometric parameters included BMI, age, and gender. Blood parameters included HbA1c, fasting blood glucose (BG), cholesterol, fasting insulin, triglycerides (TG), HDL, non-HDL, LDL, ratio of cholesterol to HDL (CHO/HDL), VLDL, and homeostatic model assessment for insulin resistance (HOMA-IR). We also included baseline glucose at the start of the meal as a predictor. Following Zeevi *et al*.^25^, we computed the 2 hour iAUC for each PPGR recorded using the Abbott FreeStyle Libre Pro (results on the Dexcom G6 Pro device are comparable, and thus not reported). Following Zeevi *et al*.^25^ as well, we used an extreme gradient boosting (XGBoost)^26^ model with 80 tree estimators, max depth of 1, learning rate of 0.2, L1 regularization of 1, and no L2 regularization to predict iAUC and AUC from those features. Using a leave one subject out procedure (i.e., train on data from 44 participants, test on the remaining participant), we obtained a Pearson correlation of 0.89 (*p* < 0.001) between ground truth and predicted AUC; see Fig. 4a. A separate XGBoost model predicts iAUC with a correlation of *ρ* = 0.64 (*p* < 0.001) with respect to ground-truth iAUC; see Fig. 4b. These correlations between predicted and actual iAUC in our dataset are consistent with those reported by Zeevi *et al*. on a cohort of 800 participants (*ρ* = 0.68) and a separate validation cohort of 100 participants (0.70)^25^ and Mendes-Soares *et al*. (*ρ* = 0.62)^27^ on a different cohort with 327 participants, which support the validity of the CGMacros dataset^22^. It is notable that, despite having an order of magnitude fewer participants, predictions from CGMacros are similar to those reported on those studies, which adds further credence to the validity of the CGMacros dataset^22^.

**Fig. 5:**
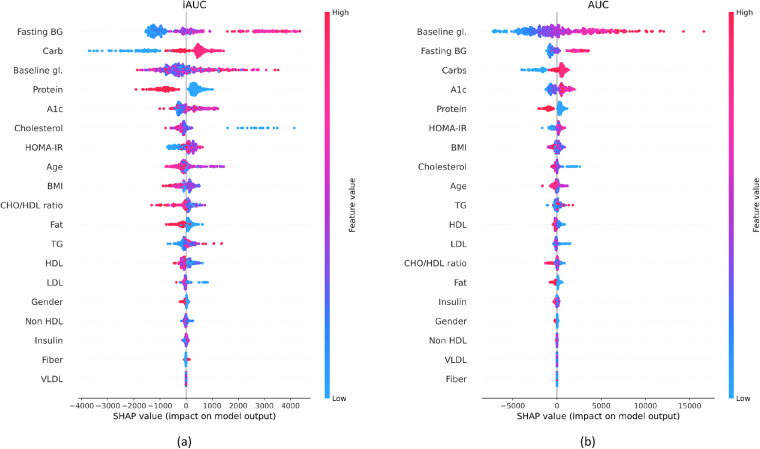
Beeswarm plot of SHAP values for (**a**) iAUC and (**b**) AUC predictions. Features are ranked from highest (top) to lowest (bottom). Each point represents an instance (i.e., a meal for a participant), color coded by the feature’s value (red: high; blue: low) on that instance and its horizontal position representing its impact on the corresponding AUC/iAUC (right: positive; left: negative). For example, high carbohydrates (red color) have a positive (right side) contribution to iAUC.

In a final analysis, we examined the importance of each feature when predicting AUC and iAUC. For this purpose, we trained the XGBoost model on data from all study participants, and computed the SHapley Additive exPlanations (SHAP)^28^. Figure 5 shows a beeswarm plot of the predictors’ “importance” and their relationship with the dependent variable. Predictors are ranked by their importance (from top to bottom), each point representing an instance (i.e., a meal for a given participant). Each point is color coded according to the value of the corresponding feature on that instance (red: high; blue: low), and its horizontal position denotes whether the feature leads to a higher (right) or lower (left) prediction of AUC or iAUC. Fasting glucose (measured during participant recruitment) is the strongest predictor for iAUC and the second strongest for AUC, in both cases showing a positive correlation that is consistent with the literature^29^. The amount of carbohydrates in a meal (converted into calories) is also a strong predictor for iAUC (#2) and AUC (#3), in both cases showing an expected positive correlation (i.e., carbohydrates are the main determinant of postprandial glucose). Baseline glucose (i.e., immediately prior to meal intake) is the strongest predictor for AUC, as expected, and third for iAUC, consistent with the fact that baseline glucose is subtracted when computing the iAUC. HbA1c is the fifth strongest predictor for iAUC and fourth for AUC, also with a positive effect that is consistent with findings that elevated HbA1c leads to higher postprandial glucose responses^30^. The amount of protein in the meal (again converted into calories) is the fourth strongest predictor for iAUC and fifth for AUC, with a negative correlation that is consistent with its suppressing effect on postprandial glucose^31,32^. The amount of fat in the meal also shows a negative correlation, reflecting its suppressing effect on postprandial glucose (i.e., due in part to gastric emptying), but is not a strong of a predictor (#12 for iAUC and #9 for AUC) as protein. Overall, these results agree with prior literature on the main contributors to elevated postprandial glucose, further supporting the validity of the CGMacros dataset^22^.

Beyond prediction of postprandial glucose responses to meals, CGMacros could support further research in diet monitoring. A prime example is in the development of models and algorithms to predict the macronutrient composition of meals based on postprandial glucose responses with minimal user intervention^15,16^. Such models can be thought of as the “inverse” problem^33^ of the one discussed in this section, whose goal is to predict postprandial glucose responses to meals based on their macronutrient content (i.e., the “direct” problem). CGMacros may also be used to develop “meal announcement” algorithms^34^ for closed-loop insulin delivery systems by identifying moments of dietary intake from CGM recordings. Finally, CGMacros may also be used to develop interpretable models (e.g., parametric) of the relationship between health parameters (e.g., HbA1c, lipid profiles) and macronutrients in mixed meals.

## Data Availability

The CGMacros dataset is available on figshare^22^. The code for analyzing the dataset and generating all figures are also available at https://github.com/PSI-TAMU/CGMacros.
